# Emicizumab and shared medical decision in patients living with hemophilia A

**DOI:** 10.1016/j.htct.2026.106504

**Published:** 2026-09-09

**Authors:** Nicolas Guillard, Benoît Guillet, Pascal Caillet, Laurent Ardillon, Philippe Beurrier, Vincent Cussac, Brigitte Pan-Petesch, Yoan Repesse, Estelle Leroy, Valérie Horvais, Marc Trossaërt, Sonia Prot-Labarthe

**Affiliations:** a University of Nantes, Nantes University Hospital, Pharmacy, F-44000, Nantes, France; b University of Rennes, CHU Rennes, Inserm, EHESP, Irset (Institut de Recherche en Santé, Environnement et Travail) - UMR_S 1085, Rennes, France; c Hemophilia Treatment Center, University Hospital, Rennes, France; d Department of Public Health, University Hospital of Nantes, Nantes, France; e Hemophilia Treatment Center, CRC-MHC, Tours University Hospital, Tours, France; f Hemophilia Treatment Center, CT-MHC, Angers University Hospital, Angers, France; g Hemophilia Treatment Center, Hospital of Le Mans, Le Mans, France; h Hemophilia Treatment Center, Morvan University Hospital, Brest, France; i Hematology Laboratory and Hemophilia Reference Center, Caen University Hospital, Caen, France; j Nantes Université, CHU Nantes, Unité d’Investigation Clinique Biologie, F-44000, Nantes, France; k Hemophilia Treatment Center, Nantes University Hospital, Nantes, France; l INSERM UMR 1246, SPHERE: Methods in Patient-Centered Outcomes and Health Research, Nantes, France

**Keywords:** Shared medical decision, Shared decision making, Hemophilia, Emicizumab

## Abstract

**Aim:**

Emicizumab is a new prophylactic treatment for patients with severe hemophilia A. This study evaluated patient decisional conflict and physician decisional comfort regarding the switch to emicizumab, identifying the criteria that drive this choice.

**Methods:**

This is an observational, non-interventional, multicenter, retrospective and exploratory study of hemophilia A patients at seven centers in France. The evaluation used the patient's decisional conflict scale and the physician's decisional comfort scale, as well as a questionnaire to determine the criteria for choosing whether to switch to emicizumab. A cluster approach was applied to identify patient subgroups with distinct decisional profiles.

**Results:**

A total of 53 patients and their physicians were included in this study: 34 in the emicizumab arm and 19 in the factor VIII arm. Patients in the factor VIII arm reported higher remorse, while physicians in this group felt significantly less comfortable with the decision. While factors influencing the decision were similar between both patients and physicians, illustrating good mutual understanding. Cluster analysis identified a specific subgroup (23%) in the total cohort experiencing high decisional conflict despite their final choice, highlighting a need for targeted clinical support.

**Conclusion:**

This study highlights the importance of shared decision-making in hemophilia treatment, encouraging open communication between patients and physicians for more personalized care.

## Introduction

Nowadays, the widespread availability of medical information challenges traditional patient-physician relationship models [1]. The paternalistic approach is increasingly replaced by models favoring patient autonomy and collaboration. To promote this shift, the French Health Authority published guidelines in 2013, titled "Patient and healthcare professionals: deciding together (Concept, aids for patients and the impact of shared medical decision-making)" [2]. Consequently, various shared decision-making (SDM) tools have emerged across therapeutic areas to help clarify options, health outcomes, and personal values [3]. These instruments assess critical dimensions such as decisional conflict, preparation, and potential regret.

Severe hemophilia A (HA) is an X-linked inherited bleeding disorder caused by a deficiency in coagulation factor VIII (FVIII). The prevalence of the disease is approximately 1 in 20,000 male births. While management has historically relied on intravenous FVIII concentrates, the development of FVIII inhibitors remains a major complication. Recent decades have seen significant therapeutic breakthroughs, including long-acting drugs, mimetic agents, coagulation inhibitors and gene therapy [4]. This expanding landscape allows for personalized care tailored to bleeding phenotype, pharmacokinetic profiles and pharmacodynamic characteristics, joint health, lifestyle, adherence to treatment, personal risk of inhibitors and patients preferences [5]. Valentino et al. described the integration of SDM in Hemophilia [6]. They identified tools that could be useful, but dedicated tools remain scarce. Existing resources often focus on pediatric prophylaxis or specific choices between standard and extended half-life products [[7], [8], [9]].

Emicizumab is a new therapeutic alternative, for the management of HA. This bi-specific monoclonal antibody mimics the function of FVIII by binding to activated factor IX and factor X, thus restoring effective hemostasis in patients with HA [10,11]. Unlike traditional factors, emicizumab has been available through both hospital and retail pharmacies in France since June 2021 [12,13]. Its subcutaneous administration and flexible dosing schedules offer substantial quality-of-life benefits [14]. However, transitioning to emicizumab alters the patient’s care routine and clinical monitoring, as it interferes with standard coagulation tests like activated partial thromboplastin time (aPTT). Furthermore, emergency management requires specific protocols, such as avoiding activated prothrombin complex concentrates (aPCC) due to thrombotic risks. Given these complexities, the clinical relevance of the decision-making process is paramount. The aim of this study was to evaluate SDM between patients and physicians when initiating emicizumab therapy.

### Materials and methods

This observational, non-interventional, multicentric, retrospective and exploratory study was conducted across seven university hospitals within the BERHLINGO network (Réseau et Base d'Etude et de Recherche en Hémostase pour Les INvestigateurs du Grand-Ouest). The inclusion criteria focused on patients with HA aged over 1 year old who had made a therapeutic decision regarding emicizumab within the previous year. Patients were stratified into two groups: those who switched to emicizumab (Group S) and those who maintained their current treatment (Group NS; non-switched). The project received ethical approval from Rennes University Hospital Health Ethics Group (n° 22.40).

Data collection occurred between March 2022 and May 2023. While the initial recruitment target was 70 patients (10 per center), final enrollment reached 53 patients. This sample size was prioritized to maintain high data quality and minimize recall bias, ensuring that the participants' reflections on their decision-making process remained accurate and contemporary. Decision conflict and comfort were evaluated using scales adapted from Legaré et al. for patients and physicians, respectively [15,16]. Additionally, a specific questionnaire was drawn up to determine the criteria influencing the therapeutic choice, including clinical factors (severity, inhibitors), environmental influences (medical motivation, patient associations), and treatment-related appeal (administration route, autonomy). Patient and caregiver independently completed two iterations of the same instrument.

The statistical analysis was conducted in two stages. First, quantitative and qualitative variables were compared between groups using Student’s t-tests or Fisher’s exact tests, respectively with statistical significance adjusted via Bonferroni correction for multiple comparisons.

Second, a cluster analysis was performed to identify homogeneous patient profiles. Data were synthesized into a matrix using Gower's distance after discretizing quantitative variables into quartiles or quintiles. A hierarchical cluster algorithm tested both bottom-up and top-down methods across four models. The optimal number of profiles was determined using the elbow method, silhouette method and cluster size. This subgroup analysis was specifically designed to identify clinical sub-profiles that may require targeted support in a SDM context, thereby bridging the gap between statistical findings and clinical practice. Once completed, a descriptive approach was adopted to describe the profiles using the same method as the general approach.

## Results

**Fig. 1:**
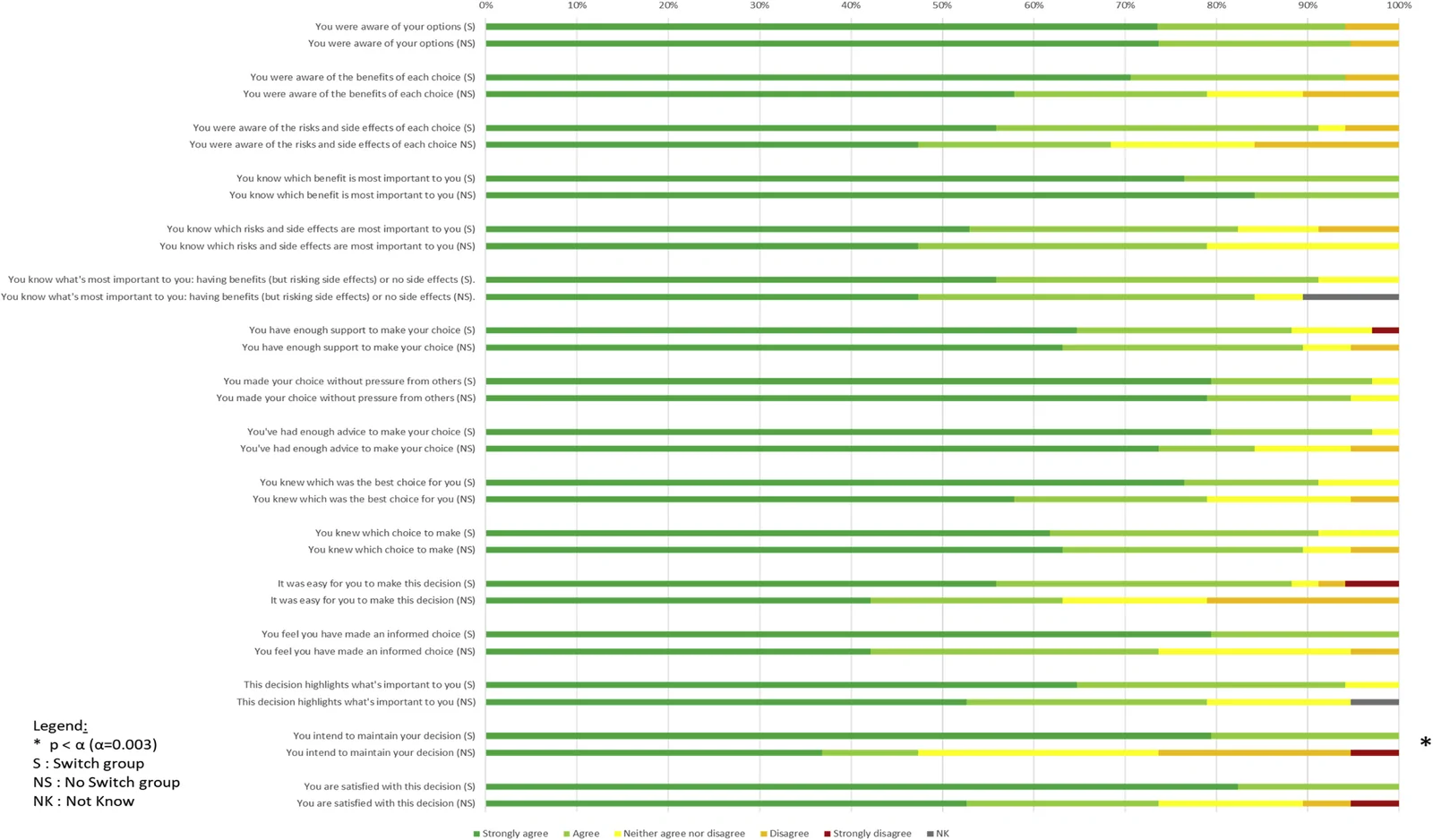
Scores from the Patient Decisional Conflict Scale (adapted from Legaré et al. [16]). The cohort comprised 34 patients in the Switched group and 19 patients in the Non-Switched group

The study included 53 patients from seven hemophilia treatment centers allocated either to the S Group (n = 34; 64%) or the NS Group (n = 19; 36%). There was no significant difference in patients’ characteristics between the two cohorts (Table 1). Regarding the patient’s decisional conflict scale (Figure 1), the S Group showed a significantly higher frequency for maintaining their decision compared to the NS Group.

**Table 1:** Patient characteristics.

Variable	Answers	Group S (n = 34)	Group NS (n = 19)	Total (n = 53)
Male sex° - n (%)		34 (100.0)	19 (100.0)	53 (100.0)
Median age* (years) – median (range)		41 (13-73)	41 (19-72)	41 (13-73)
Hemophilia - n (%)	Moderate°	1 (2.9)	0 (0.0)	1 (1.9)
	Severe°	33 (97.1)	19 (100.0)	52 (98.1)
HTRC - n (%)	Angers°	3 (8.8)	2 (10.5)	5 (9.4)
	Brest°	3 (8.8)	1 (5.3)	4 (7.5)
	Caen	5 (9.4)	0 (0.0)	5 (9.4)
	Le Mans°	4 (7.5)	4 (21.1)	8 (15.1)
	Nantes°	5 (9.4)	5 (26.3)	10 (18.9)
	Rennes°	5 (9.4)	5 (26.3)	10 (18.9)
	Tours°	9 (17.0)	2 (10.5)	11 (20.8)
Treatment at time of decision - n (%)	Efmoroctocog alfa (Elocta®) for prophylaxis°	17 (50.0)	11 (57.9)	27 (50.9)
	Efmoroctocog alfa (Elocta®) on demand°	4 (11.8)	2 (10.5)	6 (11.3)
	Octocog alfa (Kovaltry®) for prophylaxis°	4 (11.8)	2 (10.5)	6 (11.3)
	Lonoctocog alfa (Afstyla®) for prophylaxis°	3 (8.8)	0 (0.0)	3 (5.7)
	Octocog alfa (Advate®) for prophylaxis°	2 (5.9)	1 (5.3)	3 (5.7)
	Moroctocog alfa (Refacto®) for prophylaxis°	1 (2.9)	1 (5.3)	2 (3.8)
	No treatment°	1 (2.9)	0 (0.0)	0 (0.0)
	Others for prophylaxis°^1^	0 (0.0)	1 (5.3)	1 (1.9)
	Other on demand°^2^	2 (5.9)	1 (5.3)	3 (7.5)
Injection before/after shared decision-making - n (%)	Self-injecstion °	28 (82.3) / 30 (88.2)	17 (89.5) / 17 (89.5)	45 (84.9) / 47 (88.7)
	Nurse°	5 (14.7) / 4 (11.8)	1 (5.3) / 1 (5.3)	6 (13.2) / 5 (9.4)
	Family caregiver and/or nurse°	1 (2.9) / 0 (0.0)	1 (5.3) / 1 (5.3)	2 (3.8) / 1 (1.9)
Injection place before/after shared decision making	At home° - n (%)	31 (91.2) / 32 (94.1)	19 (35.8) / 19 (35.8)	50 (94.3) / 51 (96.2)
	Hospital° - n (%)	3 (8.8) / 2 (5.9)	0 (0.0) / 0 (0.0)	3 (5.7) / 2 (3.8)
	Distance from home to nearest HP* (in km) – median (range)	10 (1-170)	10 (2-34)	10 (1-170)

1 Novoeight® in Group NS (n = 1; 1.9%)

2 Novoeight® in Group NS (n = 1; 1.9%), Advate® in Group S (n = 1; 1.9%) and Feiba® in Group S (n = 1; 1.9%);

° percentages calculated in relation to the number of patients included in each group and in total

⁎ quantitative variable described as median (minimum - maximum); HTRC: Hemophilia Treatment Reference Center; HP: Hospital Pharmacy; NS Group: Did not Switch to emicizumab; S Group: Switch to emicizumab

**Fig. 2:**
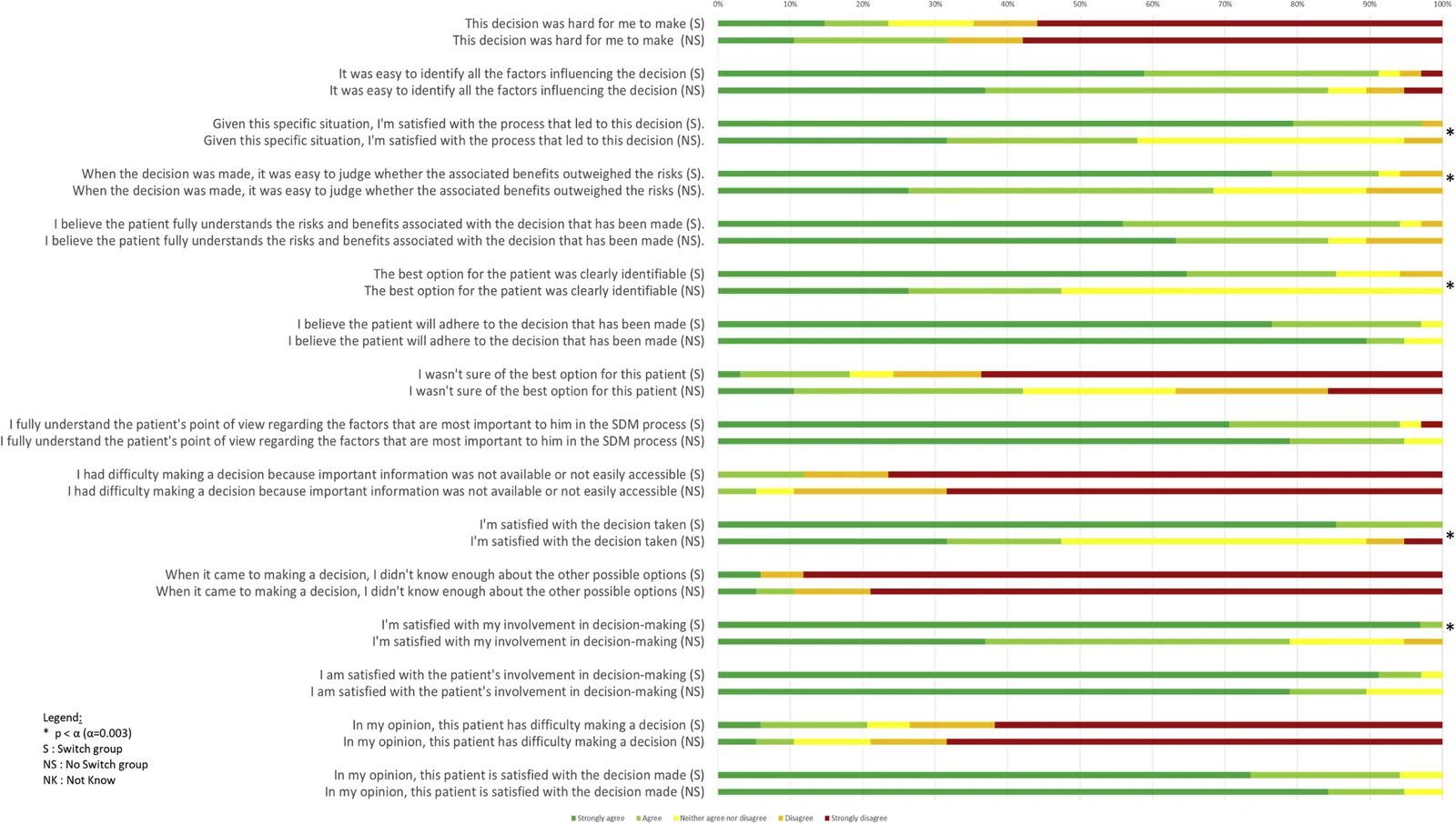
Scores from the physician's decisional comfort scale (adapted from the Ottawa Hospital decisional conflict scale) The cohort comprised 34 patients in the Switched group (S) and 19 patients in the Non-Switched group

The physician’s decisional comfort also differed significantly (Figure 2). Doctors of S Group patients reported higher satisfaction across four key items: “given this specific situation, I am satisfied with the process that led to this decision”, “when the decision was made, it was easy to judge whether the associated benefits outweighed the risks”, “I am satisfied with the decision that was made” and “I am satisfied with my involvement in the decision-making process”.

An analysis of treatment selection criteria (Table 2) revealed no significant discrepancies between patients and their physicians, suggesting strong concordance within these patient-physician pairs. This alignment is further supported by the qualitative free comments collected from both parties (Table 3, Table 4).

**Table 2:** Decision criteria influencing treatment modification in 53 physician-patient pairs, stratified by Switched (S - n = 34) and Non-Switched (NS - n = 19) Groups.

Choice criteria for receiving / *Not receiving* emicizumab	S Group (%)			NS Group (%)		
	Physician	Patient	p-value	Physician	Patient	p-value
Need / *No need* to change treatment	3 (8.8)	7 (20.6)	0.305	12 (63.2)	9 (47.4)	0.305
Desire / *No desire* to change treatment	10 (29.4)	12 (35.3)	0.796	11 (57.9)	11 (57.9)	0.796
High / *low* motivation felt at medical or nursing level	14 (41.2)	19 (55.9)	0.332	1 (5.3)	0 (0.0)	1.000
Preference for / *difficulty with* subcutaneous injection	31 (91.2)	25 (73.5)	0.109	1 (5.3)	3 (15.8)	1.000
Fear of inhibitors	1 (2.9)	0 (0.0)	1.000	0 (0.0)	1 (5.3)	1.000
Experiences in the environment that did / *didn't* motivate him/her	9 (26.5)	8 (23.5)	1.000	1 (5.3)	2 (10.5)	1.000
Patients' association made him want to / *NA*	6 (17.6)	5 (14.7)	1.000	NA	NA	NA
NA/ *Lack of feedback on this new drug*	NA	NA	NA	7 (36.8)	6 (31.6)	0.332
NA/ *Lack of confidence in the new drug*	NA	NA	NA	5 (26.3)	3 (15.8)	0.109

(S group - question in bold; NS group - question in italics)

NA: Not applicable

**Table 3:** Representative qualitative feedback from 20 of the 34 patient-physician pairs regarding factors influencing the transition to emicizumab (Switch group).

Patient	Physician
Less injections	-
Quality of life, simplicity	-
Expected benefits for joints	Fear of arthropathy in an additional joint
General research therapeutics	No previous prophylaxis ("refusal" by the patient, who did not understand its usefulness)
-	Hope for reduced joint pain
Confidence in the doctor and nurses for this new treatment	Persistent joint pain despite prophylaxis
Improve my situation after many years of stagnation	Hoped for better pain relief
Better protection, simplicity, one injection every 15 days	-
Sports without bleeding accidents	Sports practice
Frequency of injections	-
-	Discussion of treatment during clinical trials with HTRC physicians
Feeling of greater basic security	Better hemostatic disease control, better compliance likely
Switching to one injection instead of three is cool	Better compliance and therefore better control of hemophilia expected
Family pressure	-
When I was forced to take Kardegic® (stroke), I had to infuse factor VIII regularly. This requirement was a determining factor in my decision to consider treatment with Hemlibra® to protect against possible bleeding from Aspirin®.	-
Constant level of protection	-
-	Need for prophylaxis and no effective alternative in case of inhibitor
-	Hospitalization with the appearance of an inhibitory antibody (low level) with factor VIII assays lower than expected; this possibility (of switch) had previously been discussed but not accepted
Reduce treatment frequency, make foreign vacations easier. Sure to succeed and not miss the vein	Better feeling of being protected and, above all, less impact on activities, profession... with the frequency of injections
-	Discussion for months to get patient and wife to think things over. Difficulties of IV injections by himself. Did not want to change treatment

HTRC: Hemophilia treatment reference center

**Table 4:** Representative qualitative feedback from 7 of the 19 patient-physician pairs regarding factors influencing the choice not to transition to emicizumab (Non-switch Group).

Patient	Physician
Prophylaxis with Elocta® suits me (2 injections per week)	-
-	Would like to keep Elocta® prophylaxis because it is taken before weekly sports activities
Work incompatible with frequent visits to HTRC to start Hemlibra®	No time to change at the moment (very busy professional schedule) but he would like to have Hemlibra® soon
Unavailable due to work	Difficulty understanding "how such a drug can work" => NO TRUST
Lacks flexibility. Doesn't adapt to someone who lives more than an hour away	-
-	Waiting for a new treatment
Four first injections at the university hospital, a constraint regarding work	Unable to free up time for first four weekly injections at HTRC (distance +++, work +++) otherwise he would agree

HTRC: Hemophilia treatment reference center

The subgroup analysis identified a total of 12/53 (23%) patients whose responses significantly deviated from the rest of their respective groups. Interestingly, their baseline characteristics (ages, hemophilia severity, location, type of treatment, location of injection and person performing the injection) were not significantly different from other patients.

**Fig. 3:**
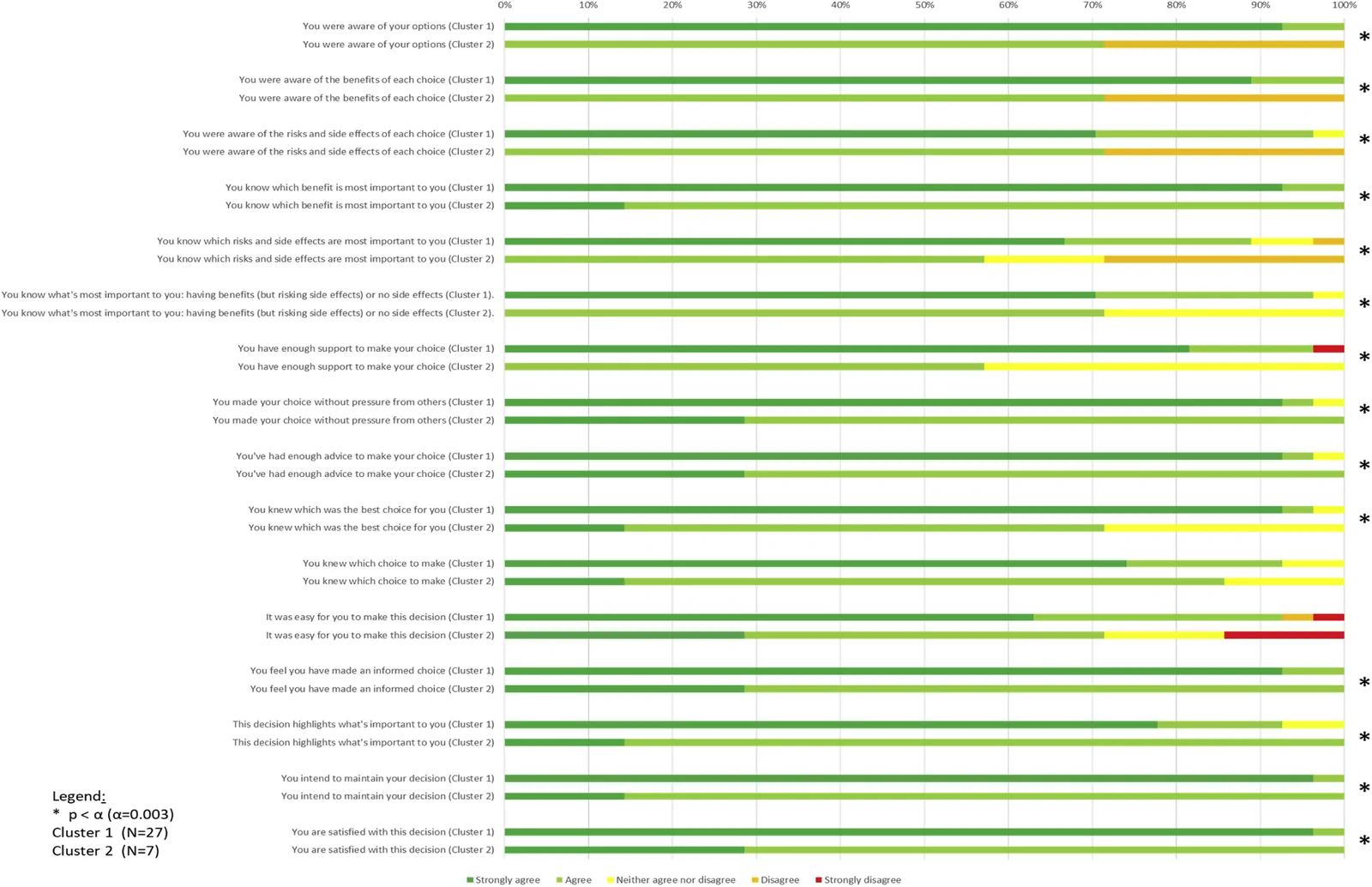
Responses to the decisional conflict scale of the 34 patients in the Switch Group divided into clusters

**Fig. 4:**
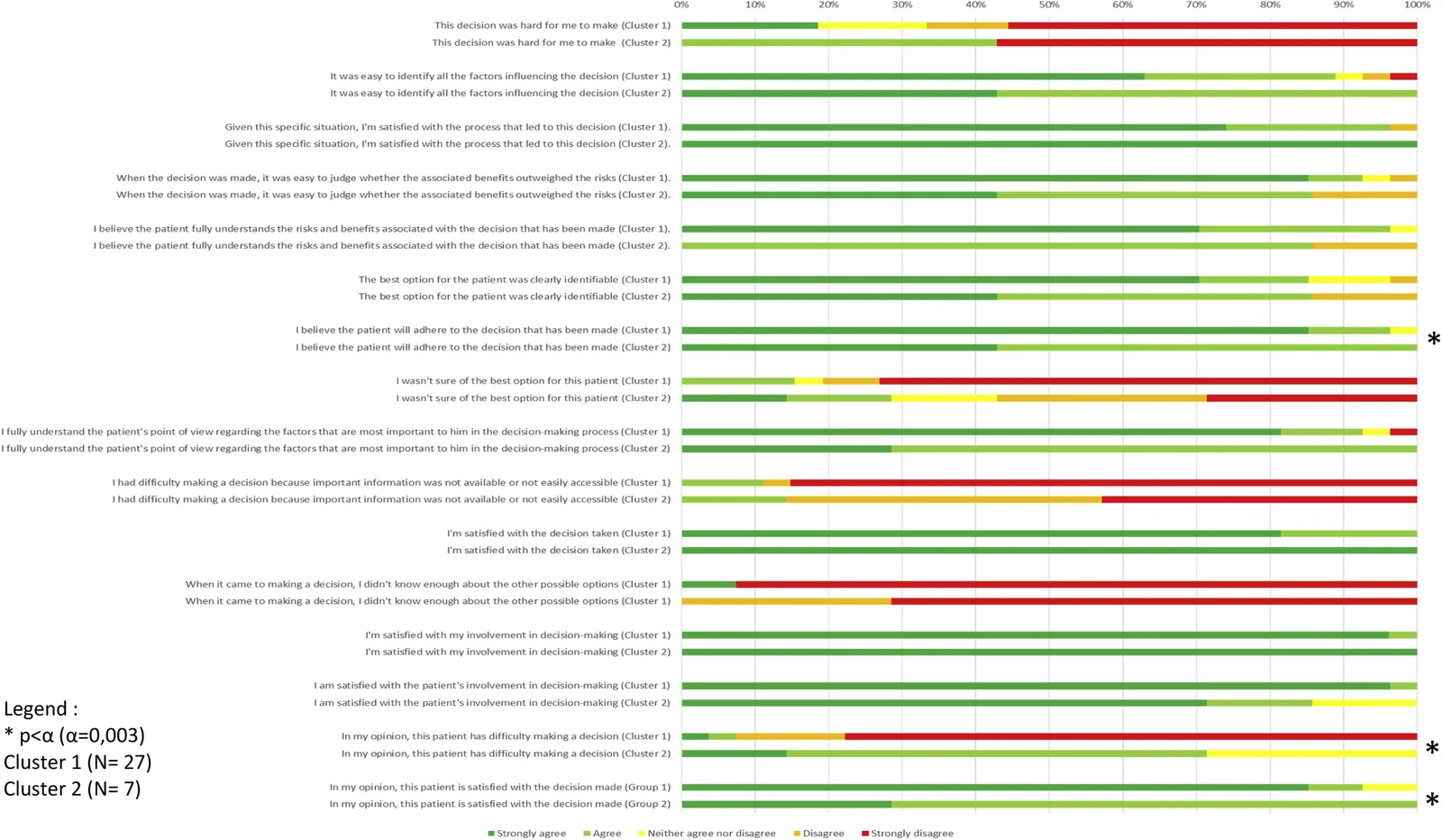
Responses to the physician's decision comfort scale for the 34 patients in the Switch Group S divided into clusters

The S Group included 7/34 (20%) of these patients. Fig. 3, Fig. 4 show the responses for which significant differences were found between this subgroup and the other patients. Figure 3 details the results of the Patient Decisional Conflict Scale, while Figure 4 shows those of the physician decisional comfort scale. This subgroup of patients (cluster 2) experiences greater decisional conflict than the remainder of the group on 14 of the 16 scale items (Figure 3). However, treatment selection factors did not differ between this patient cluster and the others.

**Fig. 5:**
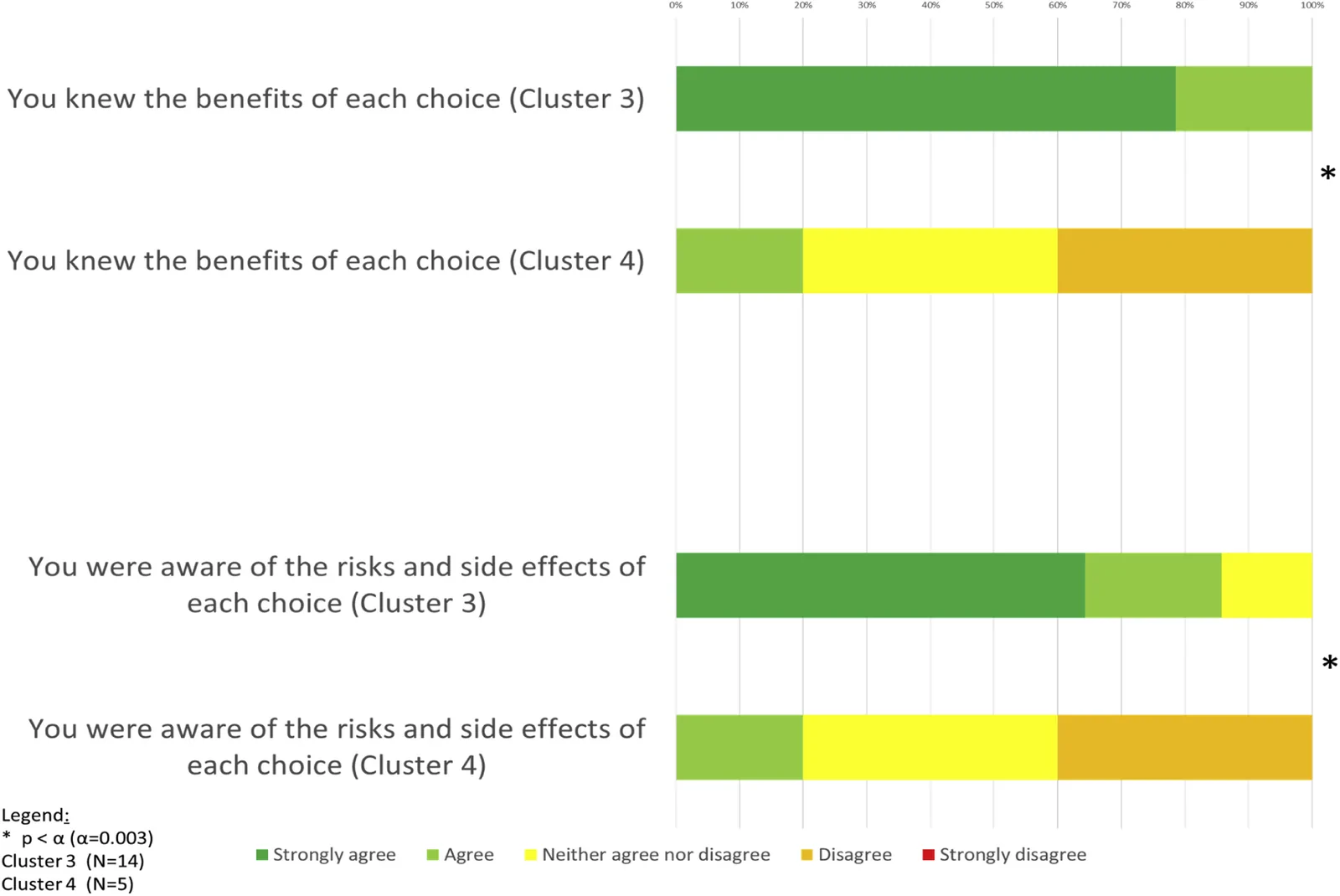
Decisional conflict scale responses of the 19 patients in the Non-switch group divided into clusters. Only the significant results are shown

For the NS Group, without change of treatment, the subgroup analysis also revealed a cluster of 5/19 (26%) patients. Figure 5 shows the results of the patient decisional conflict scale, where significant differences were observed between this subgroup and the rest of the group. Members of this subgroup perceived a lack of overall comprehension regarding the benefits and risks linked with each available option. On the other hand, there were no significant differences in the physician's decision comfort scale. In terms of factors influencing choice, only the factor "lack of hindsight on this new treatment" was found to be significantly different, and this was true for both parties involved in SDM, i.e. the physicians and the patients.

## Discussion

To our knowledge, this is the first multicenter study to gather and compare SDM data from both patients with hemophilia and their physicians. The SDM concerned here the decision to switch to prophylaxis with emicizumab versus not switching in a cohort of 53 patients from seven different hemophilia treatment centers in western France. The results demonstrate that while overall communication and understanding are high, significant differences exist in treatment maintenance and physician comfort between those who switched to emicizumab and those who did not. However, factors influencing the decision were not different between patients and physicians. Furthermore, the subgroup analysis identified a particular group of 12 patients (seven in the S Group and five in the NS Group) for whom responses were significantly different from the others in the respective cohorts. There are no other comparable publications in the field of hemophilia.

A potential limitation of this study is that the final sample size (n = 53) was below the initial target of 70 patients. This shortfall primarily affected the NS arm. Since the introduction of emicizumab, its rapid adoption in France has made identifying patients who declined this treatment within the last year increasingly difficult. To mitigate recall bias, the study limited the observation period to 12 months following the treatment decision; nonetheless, some degree of recall bias may persist as the evolution since the decision inevitably has an impact on recall [[17], [18], [19]]. Despite the smaller sample size, the multicentric design and the use of the Bonferroni correction for statistical significance ensure that the findings remain robust and externally valid.

The decision to switch to emicizumab is multifactorial, balancing medical needs with quality-of-life preferences. The route of administration was a predominant factor. The easier subcutaneous administration of emicizumab was emphasized by all participants, with particular attention paid to it by physicians. This method of administration was perceived as more accessible and less intrusive, contributing to a better quality of life for patients. This shift not only simplified personal daily management but also fostered greater patient autonomy. Although the questionnaire did not include a specific question on autonomy, this study identified a trend towards greater autonomy with emicizumab treatment. Indeed, two more patients were able to self-administer their injections after switching to emicizumab, thanks to the simplicity of subcutaneous injections.

In the patients' daily life, significant simplifications have been noted, particularly in terms of personal and professional diary management. However, it should be noted that the initiation of emicizumab is not necessarily straightforward; the requirement for frequent follow-up may act as a barrier to treatment commencement. It should be noted that some patients were considering switching to emicizumab treatment, but constraints such as the inability to attend initial patient therapeutic education sessions or a preference for other treatment options such as gene therapy hampered their decision. These factors could contribute to the feelings of conflict and remorse expressed by these patients.

Interestingly, while emicizumab was perceived as offering “continuous protection” for sports in the S Group, some patients in the NS Group felt that traditional FVIII “peaks” before physical activity were more protective. This underscores the subjective nature of safety perceptions in hemophilia care. Notably, one doctor pointed out that a patient who had refused prior prophylaxis transitioned to emicizumab highlighting the potential for this treatment to engage patients who were previously sub-optimally managed.

In conclusion, the criteria for choosing whether to switch to emicizumab treatment are multifactorial, encompassing medical, practical and quality-of-life considerations. The simplicity of subcutaneous administration, the advantages in terms of autonomy, the management of daily constraints and the possibility of accessing new patients appear to be key factors influencing the therapeutic decision.

In other clinical fields, comparable studies have examined various aspects of SDM. For example, research in orthopedic surgery evaluated SDM tools used pre- and post-operatively to reduce decisional conflict experienced by patients [20]. Another study focused on atrial fibrillation management, measuring patient satisfaction and treatment appropriateness before and after SDM. The authors concluded that SDM significantly increased patient satisfaction (25% vs. 68%, p <0.001) and treatment appropriateness (36% vs. 92%, p <0.001) [21]. Finally, Weiss et al. examined patient satisfaction in the shared decision-making process, comparing the perspectives of different healthcare professionals such as physicians, prescribing nurses and prescribing pharmacists. This study concluded that explanations on the duration of treatment correlated directly with patient satisfaction [22]. Consistently, the present study found high levels of satisfaction regarding the SDM process, with 81% of both patients and physicians reporting satisfaction with the decision-making process.

Participants in the NS Group experienced higher levels of decisional regret, as measured by the adapted Legaré et al. decisional conflict scale [15,16]. Despite our relatively small sample size and multiple variables, statistical significance was maintained applying the Bonferroni correction (adjusted α = 0.003). Notably, less than half of the patients who opted for their historical treatment expressed a desire to maintain their decision, contrasting with the unanimous commitment of those who switched to emicizumab. This regret is partly linked to physicians' perceived dissatisfaction with the decision-making process, as reflected by lower satisfaction in the NS Group regarding assessment of the decision and the balance between benefits and risks. This contrasts with physicians in the S Group, who expressed higher satisfaction and confidence. Decision regret is a well-documented phenomenon observed in various medical contexts, highlighting the intricate psychological and emotional aspects of healthcare decision-making [[24], [25], [26], [27]].

Declining a treatment change may trigger negative emotions, particularly when patients perceive missed therapeutic opportunities. The data of this study shed light on this intricate dynamic, emphasizing the significance of informed decision-making and ongoing, transparent communication between patients and healthcare professionals. These insights prompt inquiries applicable to diverse medical domains, urging additional exploration into the psychological and emotional factors shaping medical decisions and subsequent emotional responses.

This study highlights strong alignment between patients and physicians regarding decision-making factors, including joint benefits, motivations related to sports, reduced weekly injections, and enhanced disease control with improved safety. This reflects robust communication and mutual understanding in SDM. However, nuanced differences persist, with physicians emphasizing certain aspects more than patients, such as the preference for subcutaneous over intravenous injections, while patients expressing a lack of confidence in the new treatment. The comments of physicians often reflect the backgrounds of patients, notably the presence of inhibitors. Conversely, patients emphasize their feelings about the necessity for a treatment change and the perceived engagement of their healthcare providers. These differences may arise from differing interpretations of medical information, diverse risk-benefit assessments, or individual considerations. Occasional contradictions underscore the intricate nature of SDM in healthcare, emphasizing the necessity for transparent communication to address and reconcile discrepancies.

The consistency between doctors and patients reflects a trend observed in other medical fields, where mutual understanding of motivations and expectations can be a key factor in successful decision-making [22,28]. However, there are also examples in the medical literature where significant discrepancies between doctors and patients or between healthcare professionals have been observed in other pathologies [[29], [30], [31]]. These variations underline the importance of a personalized approach to SDM, considering the specificities of each patient and each medical situation. cluster analysis identified subgroups in each arm, with about 25% of patients appearing uncomfortable with their decisions, reflected in lower satisfaction among patients and physicians. For example, the cluster of “fragile switchers” (Group S, Cluster 2) suggests that a patient’s agreement to a new therapy does not necessarily imply certainty. For these patients, additional sessions with therapeutic education nurses could resolve lingering doubts and improve long-term treatment adherence. Recognizing these clusters suggests using questionnaires during decision-making could aid in providing targeted support.

The use of validated tools strengthened the credibility and robustness of this study [23,[32], [33], [34]]. However, certain limitations should be highlighted. First, an existing questionnaire was adapted for use from a retrospective perspective. This approach may have influenced participants' responses, due to the time gap between the decision and the solicitation of their opinions.

Another avenue to enrich our understanding would have been to include questionnaires for other healthcare professionals involved in patient monitoring, particularly nurses specializing in therapeutic patient education, whose perspectives could have provided additional insights.

Despite the relatively small sample size, this study offers valuable insights into SDM in personalized health and provides a basis for future research in this area. Further studies are warranted, particularly in the evolving context of therapeutic choices, such as the emergence of gene therapy, to better support patients in their decision-making.

## Conclusion

This study presents several strengths in terms of organization and methodology, notably its multicenter design involving seven different centers, which provides a broad perspective on SDM in hemophilia. By involving both physicians and patients, the study provides an in-depth understanding of SDM-related decisions. This study characterized the decision-making dynamics process surrounding the switch to emicizumab treatment. Given that emicizumab allows for flexibility in starting or returning to previous treatments, patients benefit from ongoing support to manage potential decisional regret. Furthermore, this study demonstrates a high degree of homogeneity and concordance between patient and physician experiences, particularly regarding the criteria for initiating emicizumab or not. But it also highlights some differences in the way doctors and patients felt about each other. A key contribution of this research is the identification of patient clusters that harbor hidden decisional conflict or regret. These results emphasize that patients require ongoing support, particularly those in the NS Group or those identified as “fragile switchers.” Future research could include more multidisciplinary projects, evaluation of decision aids for emicizumab, and larger-scale data collection.

### Practice implications

This study highlights the importance of SDM in the rapidly evolving landscape of hemophilia treatment. The identification of specific patient subgroups through cluster analysis suggests that a standardized counseling approach is insufficient. It also shows that the results obtained may be sensitive to contextual and individual nuances. These findings contribute to a better understanding of the factors influencing patients' therapeutic choices and reinforce the importance of open communication and close cooperation between patients and healthcare professionals to ensure that therapeutic choices align with both medical requirements and individual life goals.

## Conflicts of interest

The authors declare no conflicts of interest.

## Data Availability

The data that support the findings of this study are available from the corresponding author upon reasonable request.
